# Risk Factors Associated with Passengers with Imported Dengue Fever at International Airports in Taiwan

**DOI:** 10.3390/ijerph191711096

**Published:** 2022-09-05

**Authors:** Ying-Yun Wang, Pei-Kwei Tsay

**Affiliations:** 1 Graduate Institute of Clinical Medical Sciences, Chang Gung University, Taoyuan 333, Taiwan; 2 Department of Public Health and Center of Biostatistics, College of Medicine, Chang Gung University, Taoyuan 333, Taiwan

**Keywords:** imported dengue fever, quarantine, risk factor

## Abstract

Dengue fever (DF) is a mosquito-borne disease prevalent in the tropics (e.g., sub-Saharan Africa, Asia, and Central and South America) and a common cause of febrile illness in travelers. The high incidence of imported DF in Taiwan has led to a domestic outbreak. This study explored the risk factors associated with individuals given diagnoses of imported DF at international airports in Taiwan. The results may serve as a reference for DF prevention. In this retrospective study, data from the symptom notification system database of the Taiwan Centers for Disease Control (TCDC) were used. These data concerned travelers who returned to Taiwan from DF-endemic areas with suspected DF symptoms. The epidemiological characteristics of the cases were analyzed, and 28 variables related to DF infection were included in the multivariate logistic regression analysis. In 2018–2019, there were 8656 cases (451 positive and 8205 negative cases). The results revealed DF symptoms and a 16–30-day stay in endemic areas to be independent risk factors and the presence of three respiratory symptoms and <10 days of short-term travel to be protective factors. These results may enable the accurate assessment of symptoms in travelers with DF as well as the risk factors associated with imported DF, lowering the risk of indigenous DF outbreaks caused by imported DF.

## 1. Introduction

Dengue fever (DF) is an acute viral disease caused by four forms of serotype dengue virus (DENV), and it is spread among humans by Aedes aegypti (yellow fever mosquitoes) and Aedes albopictus (Asian tiger mosquitoes). According to a 2022 report by the World Health Organization, cases of DF have risen sharply, and half of the global population has been exposed to the risk of DF infection. Moreover, 100–400 million people are infected with DF annually [1]. DF infection primarily occurs in tropical and subtropical countries, including those in Asia, Central and South America, Africa, and northern Australia. The most severe DF pandemics were reported in Southeast Asia and the West Pacific. Taiwan, which is situated in the Pacific region, is a breeding ground for DF because of its warm and humid climate and because Southern Taiwan is widely and densely populated with A. albopictus [2]. The most severe DF outbreak in Taiwanese history occurred from 2014 to 2015, at which time more than 10,000 people were given a DF diagnosis and 45% and 52% of the cases were concentrated in Kaohsiung City and Tainan City, respectively [3]. Imported DF indirectly caused the domestic outbreak, which threatened the community pandemic prevention system [4,5]. Taiwanese people frequently conduct business or travel in Southeast Asian countries, and the number of travelers infected with DF has increased annually. The number of cases of imported DF rose from 157 in 2011 to 540 in 2019 [6,7], which is a twofold increase. Of the 1596 cases of imported DF that were documented in Taiwan between 2011 and 2016, 784 resulted from the dengue isolates DENV-1 and DENV-2, which are DENV strains that occur frequently in Southeast Asia [8,9]. Approximately 10% of febrile episodes experienced by travelers in endemic areas of Asia and Central and South America are caused by DF [10]. Approximately 36% of travelers infected with DF in endemic countries have returned to Europe during the acute phase of their infection (i.e., within seven days of the symptoms surfacing) [11].

Since 2003, the TCDC has used infrared thermal imaging screening and ear temperature measurement on inbound passengers at international airports [12,13]. All inbound passengers who experience fever (i.e., tympanic temperature ≥ 38 °C) and are returning from DF endemic areas are asked about their travel history (e.g., travel location and duration), their occupation, the people they have come into contact with, and cluster activities through the Communicable Disease Survey Form. Passengers suspected of having DF symptoms undergo quarantine measures, including blood testing and health self-management, and report their passenger information through a symptom notification system. The information is then forwarded to a health self-management system. The health departments in the administrative areas that the passengers live in then track the passengers using the health self-management system. Passengers can also voluntarily report their medical and health conditions through the network [6]. The rate of positive results for tests of imported DF in Taiwan is 55–58%, and nearly half of the country’s DF diagnoses are detected at international airports [12,13,14]. To improve DF case detection efficiency and shorten the time to diagnosis notification [3,4,5,8], Taiwan’s international airports began implementing nonstructural protein 1 (NS1) rapid screening tests on 1 January 2016. Inbound passengers are quickly informed of their test results, and those who have tested positive are provided with individualized health-care instructions, mosquito nets, and mosquito repellent. Through the infectious disease information system, the health departments in the administrative areas that the passengers live in are instructed to begin the necessary pandemic prevention measures, such as contact tracing, breeding source removal, and chemical control [6], to prevent the further spread of DF and lower the risk of community infection.

Governments worldwide have striven to contain the coronavirus (COVID-19) pandemic. However, preventing the spread of other infectious diseases remains essential because the spread of such diseases can cause domestic outbreaks, which increases the burden on medical systems. Moreover, the risk factors of DF infection must be clarified to enable effective rapid screening tests for groups at high risk of contracting DF and timely provision of appropriate professional health education and protective measures. This would lower the likelihood of severe DF infection and domestic outbreaks as well as pandemic prevention costs, thereby preventing medical resource depletion. Although studies describing the distribution of DENV serotypes and geographic trajectories of infection with DF are important, in this study, we used the travel history, demographic information, and symptoms of travelers diagnosed with DF upon entry into Taiwan to enable identification of the significant risk factors of imported DF.

## 2. Materials and Methods

### 2.1. Study Design and Participants

An analysis of the data in the Taiwan National Infectious Disease Data System (TNIDSS) database, which was developed by the CDC, was conducted. The CDC has established fever screening stations in Taiwanese international airports, with infrared thermometers used to monitor the body temperatures of inbound passengers. Passengers who have an ear-measured temperature of 38 °C or higher and are suspected of having febrile illness are required to complete the Communicable Disease Survey Form, which records each passenger’s demographic information, nationality, travel history before entering Taiwan (e.g., travel locations and durations), occupation, personal medical history, symptoms, and the address and phone number of a personal contact in Taiwan. Inbound passengers who have a fever and have stayed in DF endemic areas are considered travelers with suspected DF and must undergo blood sampling (including NS1 screening) [6]. These passengers must immediately submit their data to the TNIDSS database. The serum samples of all travelers with suspected DF are sent to the CDC laboratory for DF positivity tests in which blood samples are separated for DENV identification. If the samples test for at least one of the criteria: (i.e., positive in a reverse transcription polymerase chain reaction test; positive for IgG or IgM antibodies; a ≥4 times increase in these immunoglobins in an enzyme-linked immunosorbent assay, which is used to test the specificity of dengue envelopes [6]), the samples are confirmed as indicating DF infection and regarded as DF positive.

The data obtained from the TNIDSS database concerned inbound passengers at international airports in Taiwan between 1 January 2018, and 31 December 2019, with suspected DF. These data include each the passenger’s date of entry, sex, age, nationality, country of departure, purpose of travel, and length of stay in a DF endemic country in addition to whether they exhibited symptoms of fever, chills, headache, myalgia, bone pain, fatigue, rash, cough, a sore throat, or a stuffy or runny nose. Data were obtained for 17,127 cases, which satisfied the sampling criteria. However, data from cases involving repeated notification and indeterminate test results were removed. The remaining data were debugged and corrected. Data from 8656 cases were included for analysis, with male and female travelers involved in 48.5% and 51.5% of the cases, respectively.

### 2.2. Data Collection and Analysis

To analyze the predictors of the passengers’ DF diagnoses, the following data on the passengers were gathered: demographic information (e.g., sex, age, and nationality), travel history (e.g., country of stay, length of stay in DF endemic countries, and purpose of travel), presence of DF symptoms (e.g., rash, muscle or skeletal aches, and headache), and presence of respiratory symptoms (e.g., stuffy or runny nose, cough, and sore throat). The passengers were divided into four age groups, namely ≤19, 20–39, 40–59, and ≥60 years. Four purposes of travel were defined, namely overseas business or travel, overseas work or visit to relatives, inbound business or travel, and inbound work or visit to relatives. The lengths of stay in DF endemic countries were divided into five groups, namely ≤5, 6–10, 11–15, 16–30, and ≥31 days. Microsoft Excel 2016 (Microsoft Corporation, Albuquerque, NM, USA) was employed to create a database to compile and debug the passengers’ case data. SPSS Statistics 24.0 (IBM, Armonk, NY, USA) was used to analyze the data. The variables were analyzed through descriptive statistics, and the odds ratio (OR) of each variable was determined through univariate logistic regression analysis, with *p* < 0.05 indicating statistical significance. Multivariate logistic regression was used on significant variables to determine the risk factors. A Chi-square(χ^2^) test was performed to analyze the variable differences between the passengers who tested positive and those who tested negative.

### 2.3. Ethical Statement

This study was reviewed and approved by the TCDC Institutional Review Board (IRB 107122). All patient data remained confidential.

## 3. Results

### 3.1. General Demographic Characteristics of the Samples

Between 2018 and 2019, 53,745 inbound passengers had a fever and were identified as having DF symptoms. A total of 8656 serum samples were collected, 451 and 8205 of which had positive and negative DF test results, respectively, with a positive test rate of 5.50%. The passengers aged 20–39 years exhibited the highest positive test rate (5.81%). Furthermore, the positive test rates for men and women were 4.97% and 5.43%, respectively. Foreigner and Taiwanese passengers exhibited positive test rates of 6.65% and 4.37%, respectively. Passengers who traveled to Cambodia and Burma exhibited positive test rates of 13.85% and 12.32%, respectively. Passengers who traveled for the purposes of overseas work or visit to relatives exhibited a positive test rate of 8.48%. Passengers who stayed in DF endemic countries for 16–30 and ≥31 days exhibited positive test rates of 11.56% and 7.86%, respectively. Passengers who exhibited no respiratory symptoms exhibited a positive test rate of 7.58%. Passengers with three DF symptoms exhibited a positive test rate of 9.09%. According to the univariate analysis results, DF infection was significantly correlated with passenger nationality, age, country of departure, purpose of travel, length of stay in DF endemic countries, and self-reported respiratory and DF symptoms (Table 1).

### 3.2. Multivariate Analysis of Logistic Regression

After nationality, age, and country of departure were calibrated, a multivariate logistic regression analysis was performed for the positive test rates, demographic information, purpose of travel, and symptoms. The results indicate that inbound travelers exhibited a higher correlation with DF infection than overseas travelers. Passengers who traveled for the purposes of overseas work or to visit relatives, inbound work or to visit relatives, and inbound business or travel were respectively 1.63, 1.65, and 1.68 times more likely to be infected with DF than those traveling for overseas business or travel, with this difference achieving the level of significance.

A long stay in DF endemic countries was correlated with an increased likelihood of positive test results. Compared with those who stayed in endemic countries for ≤5 days, passengers who stayed in such countries for 11–15, 16–30, and ≥31 days were significantly more vulnerable to DF infection, with adjusted ORs of 2.87, 4.35, and 2.54, respectively. However, no significant differences were identified between the passengers who stayed in the endemic countries for 6–10 and 1–5 days.

The ORs of the passengers with one, two, and three respiratory symptoms were respectively 0.54 (95% CI = 0.43–0.67), 0.33 (95% CI = 0.24–0.47), and 0.29 (95% CI = 0.17–0.49) times lower than that of passengers without respiratory symptoms. All the differences were statistically significant.

The ORs of the passengers with three, two, and one DF symptoms were respectively 3.26 (95% CI = 1.77–5.99), 1.99 (95% CI = 1.37–2.89), and 1.69 (95% CI = 1.35–2.10) times higher than that of passengers without DF symptoms (Table 2); all differences were significant. The explanatory power of the multivariate logistic regression model (r^2^) was 0.23.

## 4. Discussion

Of the passengers who tested positive for imported DF from 2018 to 2019, 81.6% departed from Indonesia, Malaysia, Vietnam, the Philippines, and Thailand. This is consistent with the findings of Chang (2015), who indicated that 81.6% of the passengers who tested positive for imported DF in Taiwan from 2008 to 2013 departed from Southeast Asian countries [15]; moreover, passengers that departed from Cambodia and Myanmar exhibited positive test rates of 18.3% and 12.3%, respectively, indicating that Southeast Asia remains a DF endemic area. Of the tested passengers included in this study, 3.5% of the 50.5% traveling for overseas business or travel tested positive. Furthermore, 7.3% of the passengers traveling to Taiwan for work (i.e., migrant workers) or to visit relatives tested positive, with a risk of DF infection that was 2.1 times as high as that of passengers traveling for overseas business or travel. This is consistent with the findings of other studies [16,17]. This possibly occurred because passengers traveling for overseas business or travel were provided with suitable accommodations, stayed in the destination countries for a relatively short period, and had already evaluated the hygienic environment of the destination and adopted necessary protective measures [13,18]. The passengers traveling for inbound business, travel, work, or to visit relatives stayed for longer periods in the endemic countries and, therefore, exhibited a risk of DF infection that was 1.7 times higher than that of passengers traveling for overseas business or travel. This study’s result is similar to the findings of Chan (2018) regarding the cases of DF reported in Taiwanese international airports from 2014 to 2017. Chan revealed that foreign passengers were more likely to be diagnosed with DF than inbound passengers; passengers traveling to visit relatives were 10.4 times as likely to be given a diagnosis of DF as those simply traveling. However, this study did not conduct a further investigation to determine whether any passengers traveling for inbound work or visit to relatives had been given a DF diagnosis prior to their entry into Taiwan.

A DF diagnosis was significantly correlated with some of the variables investigated in this study. Inbound business or travel, a long stay in endemic countries, and typical DF symptoms, such as a rash and musculoskeletal pain, were independent risk factors for DF infection. This is consistent with the findings of a study on passengers who flew from Southeast Asia to Japan between 2008 and 2014, which revealed that the passengers had stayed in the endemic countries for 14.5 days on average and 74% of the passengers with fever (i.e., ≥39 °C body temperature), rash, fatigue, headache, and joint and muscle pain tested positive for DF [19,20,21]. Because of asymptomatic infection with DF, upper respiratory symptoms are rare in DF cases. According to the WHO’s guideline [22], there are coughing symptoms in 21.5% of DF cases of nonspecific constitutional symptoms. According to the research on DF cases in Taiwan, 40% of cases have cough symptoms [12]. This study also found that upper respiratory symptoms were negatively correlated with confirmed cases, but we could not exclude DF only by upper respiratory symptoms.

The incubation period of DF is 4–10 days after a mosquito bite. Business travelers or group tourists who stayed in endemic countries for <6 days were significantly less likely to be infected with DF than were migrant workers or foreign spouses traveling to Taiwan to visit relatives and business travelers who had stayed in endemic countries for longer. The incubation period for DF is less than two weeks. DF can be mostly ruled out if the traveler or immigrant’s symptoms begin more than two weeks after they left the endemic area [23]. Most of the passengers who stayed in dengue endemic countries for ≥31 days were residents in these countries. Before they go to Taiwan for business or travel, they could have been infected and already recovered. Thus, the chance of finding a traveler with DF may not correlate with increased time in these countries. As a result, a 16–30 day stay in endemic areas showed a higher positive rate than for ≥31 days. Lin (2017) reported that the purpose of travel predicts DF infection [13]. The screening, sampling, and health education results evaluated in this study indicated that the passengers who stayed in endemic countries for ≥16 days had fever, and those with at least one DF symptom were more likely to be infected with DF than other passengers.

Of the 8656 passengers included in this study, 451 tested positive for DF, indicating a positive test rate of 5.5%. Regarding the rates of DF diagnoses among passengers with suspected DF infection at Taiwanese international airports, Wang (2018) reported a 5.2% positive test rate for imported DF between 2015 and 2017 [24], Chan (2018) reported a positive test rate of 4.7% for DF between 2014 and 2017 [18], Kuo et al. (2014) reported a positive test rate of 2.5% between 2008 and 2011 [25], and Kuan and Chang (2012) reported a positive test rate of 2.4% between 2007 and 2010 [26]. At screening stations at international airports of Taiwan, infrared screening is performed to identify inbound passengers from DF endemic areas with fever. The passengers are then asked about their travel history through the Communicable Disease Survey Form. Passengers who are determined to potentially be within the DF incubation period and who stayed in endemic areas for ≥6 days are listed as targets for quarantine and inspection. These passengers undergo NS1 rapid screening tests that have a specificity up to 90%, which could shorten the reporting time. Providing health education and mosquito repellent are mandatory and effective quarantine measures [12]. These measures enable quarantine personnel to easily identify inbound passengers with imported DF and allow clinical professionals to identify overseas travelers with potential DF infection and quickly complete examinations. Passengers who partook in short-term tours or who stayed in endemic areas for less than 1 week and who have no DF symptoms are considered low-risk groups. Preventive measures for such passengers are focused on health education, health self-management, and prompt visits to medical institutions after entry into Taiwan. These passengers are required to inform their doctors of their history of overseas travel and activities if they experience bodily discomfort and voluntarily report to local health departments. Passengers can also call 1922 to access Taiwan’s free epidemic prevention hotline. Reducing the number of tests performed on low-risk groups may lead to increased positive test rates for imported DF.

According to the WHO’s (2011) guideline: “Increased air travel and globalization of trade has significantly contributed to the introduction of all the DENV serotypes to most population centers of the world”. Strategies to control global DF cases include integrated mosquito control with community and intersectoral participation and active disease surveillance based on health information systems [22]. Taiwan is one of the few countries in the world that implements “active border surveillance” and immediate NS1 rapid screening for passengers suspected of having DF fever. The data from the other studies were travelers seeking medical treatment or hospitalization after entry in most of the other countries. Taiwan conducts information gathering through self-reported retrospective case-control studies upon entry, which is safe from recall biases. These data are input into the national information system in real time to ensure the timeliness of the data and to show the difference between this study and global studies.

More than 100,000 passengers entered Taiwan through international airports every day before 2020. Therefore, the workforce and cost required to maintain 24-h DF testing and quarantine are considerable. Wu (2018) found that fever screening stations in Taiwan can effectively screen approximately 50% of imported DF cases. Using the estimated lowest dengue infection base reproduction number of 1.33, according to the reference, 18 indigenous cases, on average, can be prevented by screening each imported case. Wu (2018) indicated that the average DF prevention and treatment cost for each case was TWD 20,533, and the average cost for setting up a fever screening station for each case was TWD 6967. According to a cost effectiveness analysis, screening for each confirmed imported DF case would save approximately TWD 538,000 on preventing indigenous DF cases [27]. Therefore, identifying inbound passengers with a high risk of DF would significantly reduce the costs of containing the domestic spread of the imported disease. According to Shepard et al., who compiled the medical burden of DF in the Americas, the median outpatient department cost was USD 472, the minimum was USD 72 in Cuba and the maximum is USD 2300 in Bermuda. The median hospital admission cost was approximately USD 1227, the cheapest country was Nicaragua (USD 306), and the United States (USD 17,803) had the highest costs [28]. The results of one study showed that the total economic burdens of DF patients who live in urban and rural areas were CNY 2549 and CNY 2139, respectively, in China [29]. 

In this study, as approximately 43% of imported DF cases were symptomatic, 66% of asymptomatic cases might become confirmed DF cases. Therefore, all passengers planning to travel to DF endemic countries especially the “overseas work or visit to relatives” and “inbound work or visit to relatives” groups, should be required to enhance anti-mosquito measures such as wearing light-colored and long-sleeved clothes and using mosquito repellent and mosquito nets. In addition, education leaflets in multiple languages should be provided to passengers with DF symptoms, such as fever, headache, and rash, upon entry so that they report to quarantine personnel in order to effectively contain imported DF.

Notably, most insect-borne diseases spread to Taiwan through passengers and goods at seaports and airports. Because global warming has led to a rise in fall and winter temperatures, the replication of viruses carried by mosquitoes has accelerated, and the scope of virus transmission has expanded, which threatens public health in Taiwan [30]. Thus, early testing and strengthening health education for high-risk DF passengers arriving from endemic areas should be implemented. If low-risk passengers have suspected DF symptoms after returning home, they should go to a doctor as soon as possible and inform the doctor of their travel history. Active monitoring is performed at the borders to enable the instant identification and quarantine of inbound passengers infected with the virus to prevent its inbound transmission. High-risk groups, composed of those who stayed in endemic countries for ≥16 days, have fever, and those presenting with more than three DF symptoms should be tested and provided with health education to improve the efficiency and reduce the overall cost of monitoring. Border quarantine measures represent a critical means of preventing and controlling the spread of infectious diseases in Taiwan.

## 5. Conclusions

This study had three limitations. Firstly, positive DF cases were identified through antigen and antibody test results. However, whether they were infected with DF while overseas travel could not be determined. Secondly, we could not comparatively evaluate the DENV serotypes and geographical trajectories and the exact locations of stay and activities for those passengers who tested positive for DF. However, these may not affect this research results. Third, for the symptoms atypical of DF, passengers with fever and headaches may have self-administered antipyretics or painkillers, preventing their infection from being immediately detected at quarantine stations, leading to an underestimation of the number of dengue infections.

In this study, the travel and symptom characteristics of inbound passengers diagnosed with imported DF in Taiwan between 2018 and 2019 were investigated to enable an empirical analysis of the risk factors of DF infection and to provide a reference for quarantine personnel to identify and test high-risk groups in a timely manner. Future studies can reference and quantify these risk factors to formulate a DF risk assessment scale to raise passengers’ DF awareness upon entry to Taiwan and to motivate the passengers to voluntarily report their infection.

## Figures and Tables

**Table 1:** Demographic information and positive test rates of passengers with DF at international airports in Taiwan (*n* = 8656).

Variable	Type	DF Negative (%)	DF Positive (%)	Positive DF Test Rate (%)	χ^2^	*p*-Value
Nationality	Taiwanese	5230 (63.7)	239 (53.0)	4.37	21.2	<0.001 **
	Foreigner	2975 (36.3)	212 (47.0)	6.65		
Sex	Male	3992 (48.7)	209 (46.3)	4.97	0.9	0.339
	Female	4213 (51.3)	242 (53.7)	5.43		
Age (years)	0–19	1405 (17.1)	62 (13.7)	4.23	8.3	0.039 *
	20–39	4490 (54.7)	277 (61.4)	5.81		
	40–59	1673 (20.4)	84 (18.6)	4.78		
	≥60	637 (7.8)	28 (6.2)	4.21		
Country of departure	Indonesia	1042 (12.7)	80 (17.7)	7.13	80.7	<0.001 **
	Malaysia	1398 (17.0)	67 (14.9)	4.57		
	Vietnam	2126 (25.9)	105 (23.3)	4.71		
	The Philippines	1055 (12.9)	71 (15.7)	6.31		
	Thailand	1443 (17.6)	45 (10.0)	3.02		
	Singapore	466 (5.7)	16 (3.5)	3.32		
	Burma	121 (1.5)	17 (3.8)	12.32		
	Cambodia	199 (2.4)	32 (7.1)	13.85		
	India	153 (1.9)	8 (1.8)	4.97		
	Other	199 (2.4)	10 (2.2)	4.78		
Purpose of travel	Overseas business or travel	4145 (50.5)	153 (33.9)	3.56	54.4	<0.001 **
	Overseas work or visit to relatives	626 (7.6)	58 (12.9)	8.48		
	Inbound work or visit to relatives	1373 (16.7)	108 (23.9)	7.29		
	Inbound business or travel	2061 (25.1)	132 (29.3)	6.02		
Length of stay in DF endemic countries (days)	1–5	1351 (16.5)	38 (8.4)	2.74	142.1	<0.001 **
	6–10	2844 (34.7)	74 (16.4)	2.54		
	11–15	712 (8.7)	57 (12.6)	7.41		
	16–30	612 (7.5)	80 (17.7)	11.56		
	≥31	2661 (32.6)	227 (46.6)	7.86		
Respiratory symptoms	0	3451 (42.1)	283 (62.7)	7.58	80.6	<0.001 **
	1	2615 (31.9)	112 (24.8)	4.11		
	2	1507 (18.4)	40 (8.9)	2.59		
	3	632 (7.7)	16 (3.5)	2.47		
DF symptoms	0	5453 (66.5)	257 (57.0)	4.50	19.2	<0.001 **
	1	2027 (24.7)	141 (31.3)	6.50		
	2	585 (7.1)	29 (8.6)	4.72		
	3	140 (1.7)	14 (3.1)	9.09		

* *p* < 0.05; ** *p* < 0.01.

**Table 2:** Correlation analysis of the positive DF test rates of passengers at international airports from 2018 to 2019 (*n* = 8656).

Variable	Type	Adjusted OR (95% CI)	*p*-Value
Purpose of travel	Overseas business or travel	1	
	Overseas work or visit to relatives	1.63 (1.14~2.34)	0.008 **
	Inbound work or visit to relatives	1.65 (1.01~2.68)	0.045 *
	Inbound business or travel	1.68 (1.09~2.59)	0.019 *
Length of stay in DF endemic countries (days)	1–5	1	
	6–10	1.00 (0.67~1.50)	0.992
	11–15	2.87 (1.84~4.48)	<0.001 **
	16–30	4.35 (2.81~6.72)	<0.001 **
	≥31	2.54 (1.68~3.86)	<0.001 **
Respiratory symptoms	0	1	
	1	0.54 (0.43~0.67)	<0.001 **
	2	0.33 (0.24~0.47)	<0.001 **
	3	0.29 (0.17~0.49)	<0.001 **
DF symptoms	0	1	
	1	1.69 (1.35~2.10)	<0.001 **
	2	1.99 (1.37~2.89)	<0.001 **
	3	3.26 (1.77~5.99)	<0.001 **

* *p* < 0.05; ** *p* < 0.01.

## Data Availability

Restrictions apply to the availability of these data. Data were obtained from TCDC and were available for use in this study with the permission of TCDC.
